# Chicago Biological Society

**Published:** 1881-05

**Authors:** 


### Society Reports.

### ARTICLE VI.

### THE CHICAGO BIOLOGICAL SOCIETY.

Stated meeting April 6, 1881. Dr. W. S. HAINES, President, in the chair.

After the minutes of the last meeting had been read by the Secretary, Dr. Roswell Park, Dr. R. Tilley brought two peculiar cases before the members. The first was a young man who had cut off the ends of first and second phalanges of his left little finger, and amputating the joint, but a large part of the muscular tissues and skin had remained intact. Dr. Tilley removed a sufficient part of the bones injured, but did not amputate the finger. The result was very satisfactory. The finger is shortened one-half inch, and a false joint formed at the point of union, but so near the natural joint as to escape observation; all the motions are preserved, and the usefulness of that finger does not seem to have been in the least diminished. The other case was a man over fifty years old, who has a perforation of the septum nasi three-eighths of an inch in diameter; supposed to be congenital. The same person has a detachment of the retina, for which there was found no cure, but Dr. Tilley having read of some cures lately accomplished by hypodermic injections of pilocarpine, has resolved to give that remedy a trial. The present difficulty has existed for a year, and the sight of the patient is very much impaired as to the left eye, which is the one affected.

### MASKED MEASLES (?) AND GERMAN MEASLES.

Dr. H. M. Starkey reported the case of a child who, some time previous, had had an attack of false croup, from which he had entirely recovered. The child now became feverish and had a coryza, and the Doctor was called. He made a diagnosis of measles, and the case seemed a well-marked one, but no eruption was present at any time during the disease. Eight days later another physician was called to treat another child of the same family who had just been taken sick with the same symptoms as the first. This physician also diagnosed masked measles. A third child came down with the disease, and Dr. Starkey attended it. It was precisely similar to the others, bronchitis and coryza were present, the temperature increased during the first four days reaching 103° and 104°, and disappeared in a couple of days. The children had been exposed to measles a few days before, and there were also cases of diphtheria across the street.

Dr. R. Park related his experience with 100 cases of measles which came under his observation during an epidemic several years ago. The incubation had generally been of four or five days. Seven cases which proved fatal succumbed to pneumonia or other lung complications. Some cases had exhibited but a slight eruption, but this could always be observed with a little care on the part of the physician or the nurse, and the fever had always lasted longer than in the cases reported by Dr. Starkey.

Dr. D. R. Brower had never seen masked measles, but he had observed cases of masked scarlatina, and thought that masked measles was very possible. He related his experience with an epidemic present on the West Side. It is an eruptive disease resembling scarlet fever and measles as to the rash, while it is very mild. His little girl, who has had the measles and scarlatina, had this peculiar disease, but another child of his escaped. He also had other cases in persons who had had scarlatina and the measles previously. It corresponds in every particular with German measles. The eruption develops all over the body at the same time. There is no trouble with the throat, and very little sickness. Drs. Jones and E. Ingals, whom he interviewed on the subject, had also met with cases of German measles. This is a contagious disease and should not be confounded with roseola, which is a non-contagious affection of the skin.

Dr. Starkey had a case of German measles among students in the college. The eruption resembled at first that of scarlatina.

There was a rise of a few degrees in the temperature, redness of the throat and fauces, and after a couple of days the rash resembled more that of measles. Patient had had this disease and also scarlatina a few years ago. No desquamation took place.

Some beautiful slides for the microscope, prepared by Dr. H. D. Schmidt, of New Orleans, were exhibited before the Society by Dr. S. V. Clevenger. There were 23 of them—some physiological, but most of them pathological representations of some of the cephalic and sympathetic ganglia; sections of the spinal cord, of the liver, the spleen, the kidney, the supra-renal capsule, and the mucous membrane of the stomach. Also lithographic illustrations of the pathological changes observed in yellow fever in the various organs. Dr. Clevenger then read the paper of the evening, which was a biographical sketch of Dr. Schmidt, and will be found in this number of the JOURNAL; after which he read various extracts from a treatise on Yellow Fever, by the same writer, and this will also appear in the JOURNAL. Before adjourning, the following motion was made and carried:

"Moved and seconded, that the Chicago Biological Society cordially indorse the work of Dr. Schmidt, and recommend its publication by subscription under its auspices; and that for all subscriptions handed to him, the treasurer be held responsible to Dr. Schmidt."

All the members present subscribed for the book.

